# Naringin prevents HIV-1 protease inhibitors-induced metabolic complications *in vivo*

**DOI:** 10.1371/journal.pone.0183355

**Published:** 2017-11-09

**Authors:** Sanelisiwe Nzuza, Sindiswa Zondi, Peter M. O. Owira

**Affiliations:** Molecular and Clinical Pharmacology Research Laboratory, Department of Pharmacology, Discipline of Pharmaceutical Science, School of Health Sciences, University of KwaZulu–Natal, Westville, Durban, South Africa; CCAC, UNITED STATES

## Abstract

**Background:**

Insulin resistance, glucose intolerance and overt diabetes are known metabolic complications associated with chronic use of HIV-Protease Inhibitors. Naringin is a grapefruit-derived flavonoid with anti-diabetic, anti-dyslipidemia, anti-inflammatory and anti-oxidant activities.

**Objectives:**

The study investigated the protective effects of naringin on glucose intolerance and impaired insulin secretion and signaling in vivo.

**Methods:**

Male Wistar rats were divided into six groups (n = 6) and were daily orally treated with distilled water {3.0 ml/kg body weight (BW)}, atazanavir (133 mg/kg BW), saquinavir (333 mg/kg BW) with or without naringin (50 mg/kg BW), respectively for 56 days. Body weights and water consumption were recorded daily. Glucose tolerance tests were carried out on day 55 of the treatment and thereafter, the rats were sacrificed by halothane overdose.

**Results:**

Atazanavir (ATV)- or saquinavir (SQV)-treated rats exhibited significant weight loss, polydipsia, elevated Fasting blood glucose (FBG), reduced Fasting Plasma Insulin (FPI) and expression of phosphorylated, Insulin Receptor Substrate-1 (IRS-1) and Akt proteins, hepatic and pancreatic glucokinase levels, and also increasing pancreatic caspase-3 and -9 as well as UCP2 protein expressions compared to controls, respectively. These effects were completely reversed by naringin treatment.

**Conclusion:**

Naringin prevents PI-induced glucose intolerance and impairment of insulin signaling and as nutritional supplement it could therefore alleviate metabolic complications associated with antiretroviral therapy.

## Introduction

Incorporation of HIV-1 Protease inhibitors (PIs) such as atazanavir, darunavir, amprenavir, indinavir, nelfinavir, ritonavir, saquinavir and tipranavir in Highly Active Antiretroviral Therapy (HAART) has significantly reduced morbidity and mortality attributable to HIV infections [1]. Chronic use of PIs, has however, led to metabolic complications related to glucose intolerance and overt type 2 diabetes [2, 3].

ATV and SQV are incorporated into HAART as second-line substitutes in the current management of HIV-1 infections [4] and are effective inhibitors of HIV aspartyl endopeptidase, which is essential for generation of mature HIV virions [3, 5]. Both agents are pharmacokinetically boosted with ritonavir [6] and have been reported to reduce virological and immunological markers in HIV patients [5, 7]. SQV was the first PI to be used in adults with progressive HIV-1 infections [8]. ATV is relatively a newer PI used as a substitute in combination therapy in adults and children infected with HIV [7].

PIs have been suggested to inhibit Glucose Transporter Protein 4 (GLUT4) activity in the skeletal muscles, the main site of insulin-stimulated glucose disposal [9–11]. Phenylalanine-like core structure of PIs attached to hydrophobic moieties are suggested to inhibit GLUT4 activity [11, 12]. PIs have further been reported to block proteasomes leading to lipodystrophy and dyslipidemia, which are indirect contributors to insulin resistance [13, 14]. Lagathu et al [15] have reported increased Reactive Oxygen Species (ROS) after 24–48 hour exposure to ritonavir in cultured adipocytes, which subsequently contributed to alterations in inflammatory mediators and adipocytokine concentrations. We have recently reported that PIs impair glucose-induced insulin secretion in pancreatic β-cell lines by increasing oxidative stress and hence reducing mitochondrial ATP production [16]. This has previously been attributed to upregulation of mitochondrial Uncoupling Protein 2 (UPC2) by PIs which collapses inner mitochondrial membrane potential leading to reduced ATP synthesis [17, 18].

Chronic exposure to PIs is reported to impair insulin signaling leading to decreased glucose uptake [13, 19]. It has been suggested that PIs inhibit IRS-1 phosphorylation, association of phosphatidylinositol 3-kinase (PI3-kinase) and/or Thr^308^/ Ser^473^-Akt and subsequently blocking translocation of GLUT4 vesicles to the plasma membrane [13, 19]. In the skeletal muscles, this inhibitory action leads to decreased glucose disposal and consequently to the development of insulin resistance [13, 20]. Cheng et al (19) reported reversal of impaired insulin signaling by a novel activator of the insulin receptor tyrosine kinase in 3T3-Ll cells and also reduced PI-induced insulin resistance *in vivo*.

Currently there are no standard treatment guidelines for PI-induced metabolic disturbances. Several pharmacological interventions including substitution of PIs have been implemented with limited clinical success [4]. Furthermore, medicinal plants are becoming more favorable as they have minimal side-effects generally associated with conventional medicines [21]. Naringin is a flavanone derived from citrus species and has been demonstrated to possess antioxidant, anti-diabetic, anti-dyslipidemic and anti-inflammatory activities [22–24]. Hypoglycemic effects of naringin have previously been demonstrated [25, 26]. We have recently reported that naringin improves both glucose intolerance and insulin resistance in non-diabetic rats [27]. This study, was therefore designed to investigate the potential role of naringin in PI-induced glucose intolerance, impaired insulin secretion and signaling *in vivo*.

## Materials and methods

### Materials

SQV and ATV were purchased from Aspen Pharmacare (South Africa). Unless otherwise stated, naringin and all other chemicals were purchased from Sigma-Aldrich Chemical (St Louis, MO). Rat insulin ELISA Assay kits were procured from Biovison (Mountain View, CA).

### Animals

Male Wistar rats (250–300 g) supplied by the Biomedical Resource Unit (B.R.U) of University of KwaZulu-Natal (UKZN), Durban, South Africa were kept in transparent plastic cages at the animal holding facility of the B.R.U. The rats were allowed to acclimatize for one week prior to commencement of the study and were maintained at room temperature of 23–25°C and a relative humidity of 55–60% throughout the study in a 12 h light/dark cycle. Study protocols were approved by the Animal Research Ethics Committee of UKZN (ethics reference number:—AREC/095/015D). The animals were handled with human care according to the guidelines of the Biomedical Research Ethics Committee.

### Experimental procedure

The rats were randomly divided into 6 groups (n = 6) with free access to standard commercial chow and drinking tap water ad libitum. Rats in group 1 (control) were orally treated once daily with 3.0 ml/kg body weight (BW) of distilled water. Group 2 and 3 similarly received 133 mg/kg BW of ATV with or without 50 mg/kg BW of naringin in distilled water, respectively. Group 4 was similarly treated with 50 mg/kg BW of naringin only while groups 5 and 6 similarly received 333 mg/kg BW of SQV with or without 50 mg/kg BW of naringin, respectively. Body weights and water consumption were recorded daily for 56 days (Table 1). On day 54 of treatment, all rats were transferred into metabolic cages and 24-h urine samples were collected, measured and stored -80°C for further analysis.

**Table 1:** Animal treatment schedule. Atazavavir (ATV), Saquinavir (SQV) and Naringin (Narg) were dissolved in distilled water and 1.0 ml containing relevant doses administered orally. BW-body weight per kg.

Groups	Designation	Distilled H_2_O (ml/kg/BW)		ATV (mg/kg/BW)	SQV (mg/kg/BW)	Narg (mg/kg/BW)
1	Control	3.0				
2	ATV			133		
3	Narg/ATV			133		50
4	Narg					50
5	SQV				333	
6	SQV/Narg				333	50

#### Glucose tolerance tests

Glucose tolerance tests (GTT) were conducted on the 55th day of treatment after an overnight fast. FBG concentrations were determined after tail pricking and analyzed by a glucometer (OneTouch Select; Lifescan inc., Milpitas, California, USA). Thereafter, the rats were intraperitoneally injected with D-glucose (3.0 g/kg BW) in normal saline and blood glucose concentrations measured at 0, 30, 60, 90 and 120 mins in all groups. The Area under the curves (AUC) were calculated from blood glucose-time plots (mmol/l) x time (min) in GTT and expressed as AUC units [28].

#### Animal sacrifice

On day 56 day of the study, all rats were sacrificed by halothane overdose (5% by volume in oxygen). Blood was collected in heparinized tubes by cardiac puncture, spun at 3000 rpm for 10 min and the separated plasma samples stored at -80°C for fasting plasma insulin measurements. Gastrocnemius muscles from each rat’s left hind limb were surgically excised, rinsed in 1.0 M Phosphate-Buffered Saline (PBS), weighed and snap frozen in liquid nitrogen and stored at -80°C for further biochemical analysis.

#### Fasting plasma insulin

Fasting plasma insulin concentrations were analyzed using the DRG Ultrasensitive Rat Insulin ELISA as per the manufacturer’s guidelines. Twenty-five microliters of standards and plasma samples were aliquoted in to the primary antibody coated wells of the ELISA plate, respectively. One hundred μl of enzyme conjugated 1X solution was added to each well and the plate was incubated on a plate shaker at 800 rpm for 2 h at room temperature. The plate was then washed 6 times with 700 μl of wash buffer solution 1X using an automatic plate washer, then 200 μl of substrate TMB was added and incubated in the dark for 15 min at room temperature. Stop solution (50 μl) was then added to each well, mixed on a shaker (700 rpm) for 5 sec and optical density read at 450 nm using EZ read 400 microplate reader (Biochrom Ltd, Cambridge, UK). Fasting plasma insulin concentrations were calculated from the standard curve, expressed as μg/l.

#### Determination of insulin resistance

Insulin resistance was calculated using the Homeostasis Model Assessment of insulin resistance (HOMA-IR) equation [29]:

Insulin Resistance $=\frac{FPI\;x\;FBG}{22.5}$, where FPI: Fasting Plasma insulin (μ IU ml/L), and FBG: Fasting Blood Glucose (mM)

#### Western blot protein analysis

Expression of phosphorylated IRS-1 and Akt proteins were determined by Western blot protein assay. Briefly, 100 mg of skeletal muscle samples were homogenized in 300 μl of ice-cold radio-immunoprecipitation assay buffer (RIPA buffer) supplemented with 0.15 M sodium chloride, 1% protease inhibitor cocktail, 1% triton X-100, 0.5% sodium deoxycholate, 0.1% Sodium Dodecyl Sulphate (SDS), 0.05 M Tris (pH 8) and centrifuged (12,000 rpm, 4°C, 20 min). The supernatant was transferred into 1.5 ml microcentrifuge tubes, kept on ice and protein content determined by Bradford method [30]. Sample protein standardized to 35 μg, was denatured in buffer (dH_2_O, 0.05 mM Tris-HCl (pH 6.8), glycerol, 10% SDS, β-mercaptoethanol, 1% bromophenol blue) at 95°C for 5 min, and then electrophoresed (200 V, 1.0 h) in Sodium Dodecyl Sulphate (SDS)- polyacrylamide gels (4% stacking, 10% resolving) using Bi-Rad compact power supplier. For the determination of the expression of pancreatic UCP-2, caspase -3 and -9 proteins 50 mg pancreatic tissues were similarly treated.

The proteins were then transferred onto a PVDF membranes using the Trans-Blot Turbo Transfer system (BioRad) (400 mA, 30 min) and then blocked with 5% non-fat dry milk (NFDM) in Tris-buffer saline (TBS-T) containing 0.5% Tween 20, dH_2_O, 20 mM Tris, 150 mM NaCl, pH 7.4) for 1.0 hour at room temperature. Thereafter, the membranes were incubated overnight at 4.0°C with primary antibody (1:1000 dilution in TBS-T) rabbit p-IRS-1/2 (Tyr 612, Santa Cruz Biotechnology), p-Akt1/2/3 (Ser 473, Santa Cruz Biotechnology), goat UCP-2 N-19 (sc-6526), rabbit anti-caspase-3 antibody (ab2302), rabbit anti-caspase-9 antibody (ab25758) (Santa Cruz Biotechnology), respectively. The membranes were further washed in TBS-T solution (3 times for 10 min) and then incubated with appropriate horseradish peroxidase-conjugated secondary antibodies (1: 1000 in TBS-T containing 0.01% SDS) for 1.0 h at room temperature then washed again in TBS-T buffer (3 times, 10 min) and visualized on an Odyssey Clx Infrared Imaging System (LI-COR Biosciences GmbH, Bad Homburg, Germany). Band intensities were quantified by LI-COR software (Image Studio Lite). The data were presented as relative band density and fold change.

#### ATP quantification

The ATP levels were measured using ATP Colorimetric/Fluorometric Assay Kit (BioVision Inc). Pancreatic tissues (10 mg) were homogenized in 100 μl of ice-cold 2.0 M perchloric acid with 10–15 passes, incubated on ice for 45 min. Samples were then centrifuged at 13 000 x g for 2 min at 4°C. Supernatants (100 μl) were then transferred to fresh tubes to each one of which 34 μl of 2.0 M KOH then added. The tubes were then centrifuged at 13 000 x g for 15 min at 4°C and supernatants collected. Fifty microliters of supernatants and ATP standards were then added to a 96-well plate followed by 50 μl of reaction mix. The microplate was gently agitated and then incubated at room temperature for 30 min protected from light. The optical densities were read at 570 nm and ATP concentrations interpolated from the standard curve and presented as nmol.

#### Glucokinase determination

Glucokinase concentrations in the liver and pancreas were determined using Mouse/Glucokinase ELISA kit as per manufacturer’s instructions. The tissues (100 mg) were homogenized in 400 μl of ice-cold lysis buffer (50 mM Tris (pH 7.6), 2.5 mM dithiothreitol, 4.0 mM EDTA, 150 mM KCl and 4.0 mM MgSO_4_) and centrifuged at 35 000 x g for 1.0 hour at 4°C. One hundred microliters of supernatants, standards or blanks were aliquoted into a 96-well plate coated with a primary antibody, thereafter incubated for 2.0 h at 37°C. After which, each well was aspirated and 100 μl of Detection Reagent A working solution added and then incubated for 1.0 h at 37°C with gentle agitation. After 3 washes with 350 μl of 1 X washing buffer, 100 μl of Detection Reagent B working solution was added to each well followed by incubation for 30 min at 37°C with gentle agitation. Each well was then aspirated and washed 5 times with 350 μl of washing buffer, then 90 μl of substrate TMB added followed by incubation for 20 min at 37°C. Reaction was stopped by adding 50 μl of stop solution to each well and optical densities read at 450 nm using EZ read 400 microplate reader (Biochrom Ltd, Cambridge, UK). The concentrations of glucokinase were calculated from the standard curve, with a sensitivity of 0.156 ng/ml.

#### Statistical analysis

GraphPad Prism Version 5.0 software (GraphPad Software Inc., La Jolla, USA) was used to analyse the data presented as mean ± Standard Deviation. Unpaired Students' t-test or One way Analysis of Variance (ANOVA) followed by a Bonferroni test for multiple group comparison was used to determine statistical significance between the groups. A p value of < 0.05 was considered as statistically significant.

## Results

### Body weights and water intake

**Fig 1:**
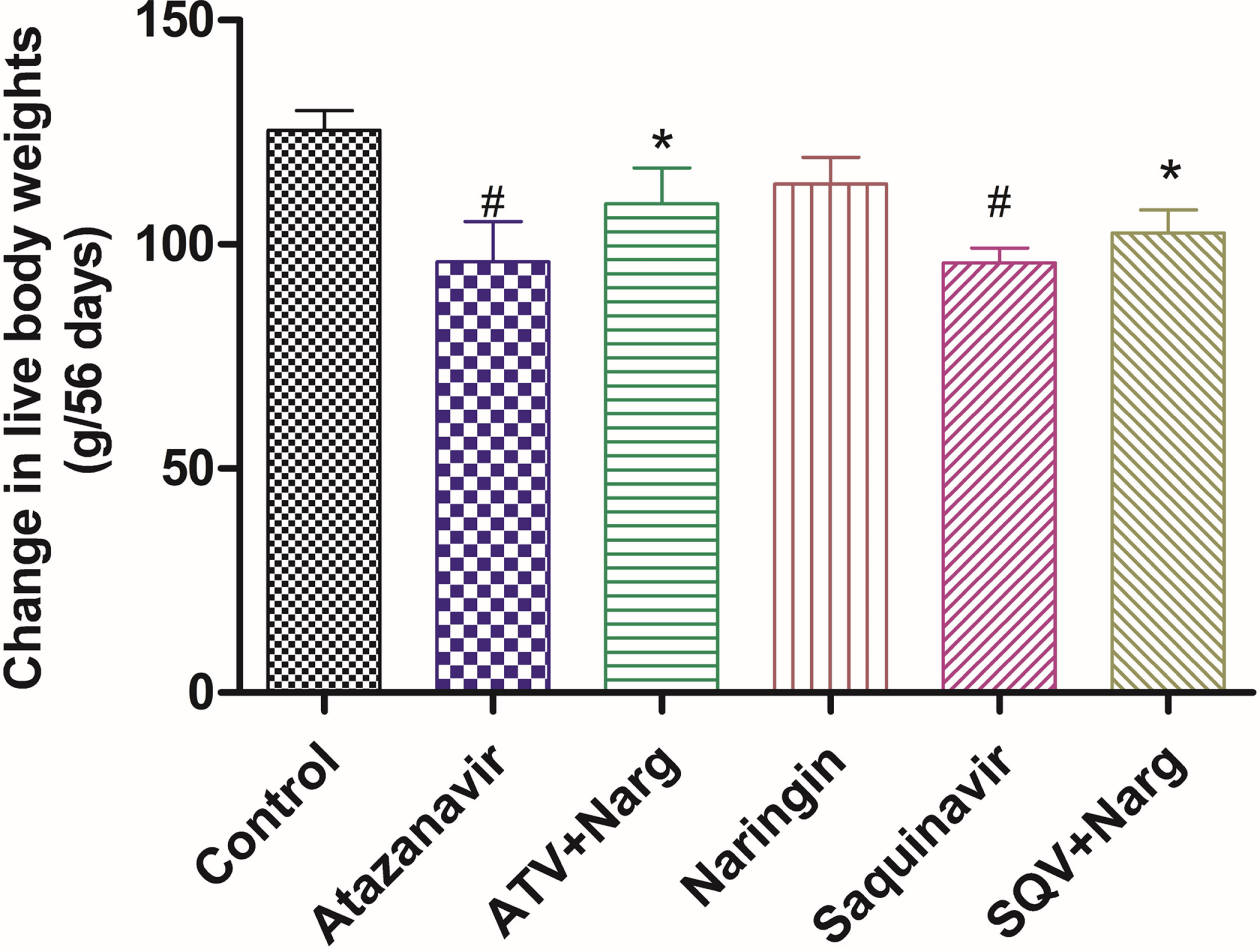
Changes in live body weights between days 0 and 56 of treatment. (^#^p = 0.0153 compared to control, * p = 0.0153 compared to ATV and SQV, respectively).

ATV- or SQV-treatment significantly (p <0.05) reduced body weights compared to control, respectively (Fig 1). However, co-administration of naringin with either ATV or SQV led to significantly (p = 0.0153) improved body weights compared to ATV- and SQV-only-treated rats, respectively (Fig 1).

**Fig 2:**
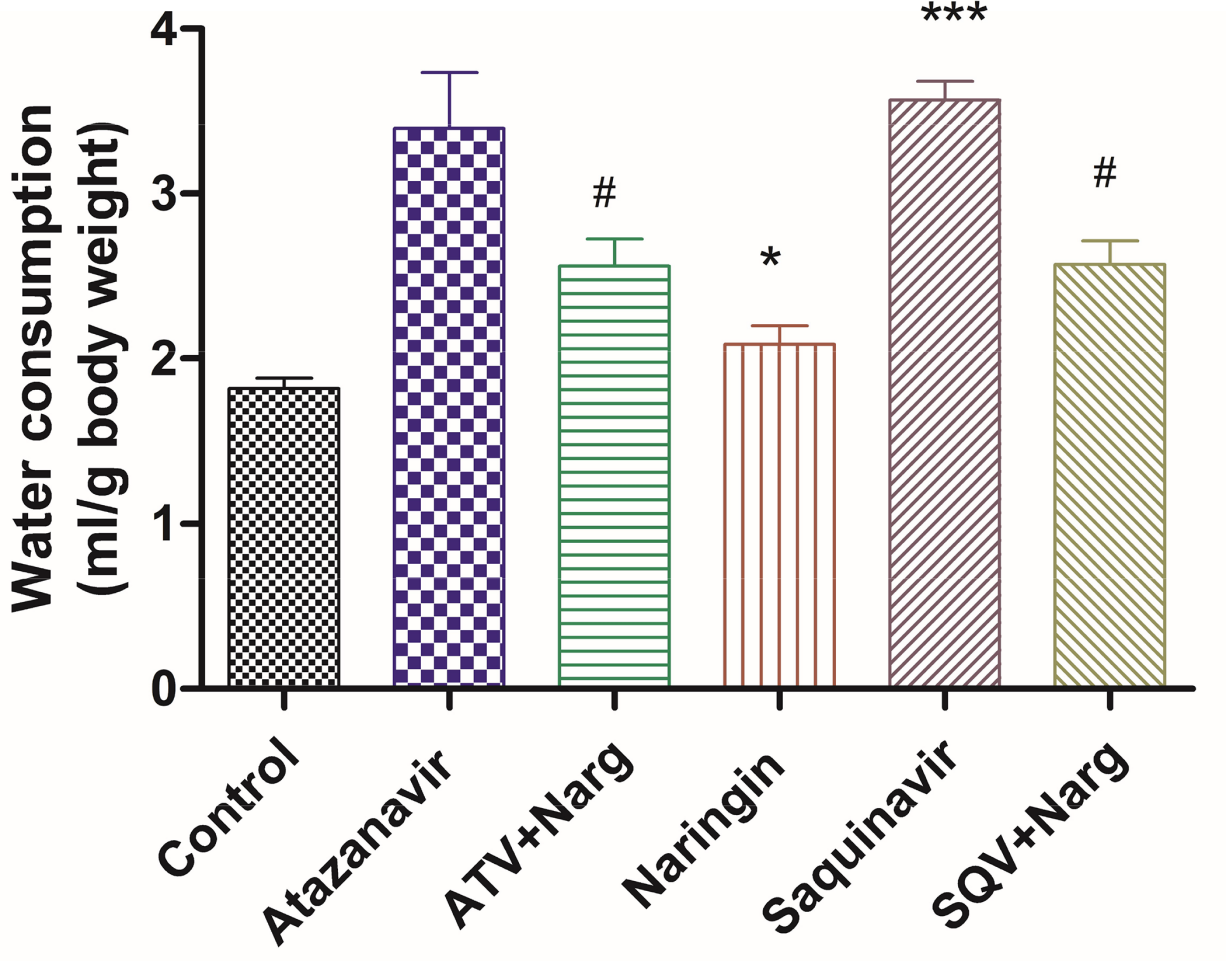
Average daily water intake per gram body weight in all treatment groups. (*, ***p < 0.05 compared to control and ^#^p < 0.05 compared to ATV or SQV, respectively).

Average daily water consumption per gram body weight was significantly (p < 0.0001) increased in ATV- or SQV-treated rats compared to the control, respectively, but co-treatment of ATV or SQV with naringin significantly (p < 0.0001) reduced water intake compared to ATV- and SQV-only treated rats (Fig 2). Naringin only-treated rats consumed significantly (p < 0.05) more water than controls (Fig 2).

### Glucose intolerance

**Fig 3:**
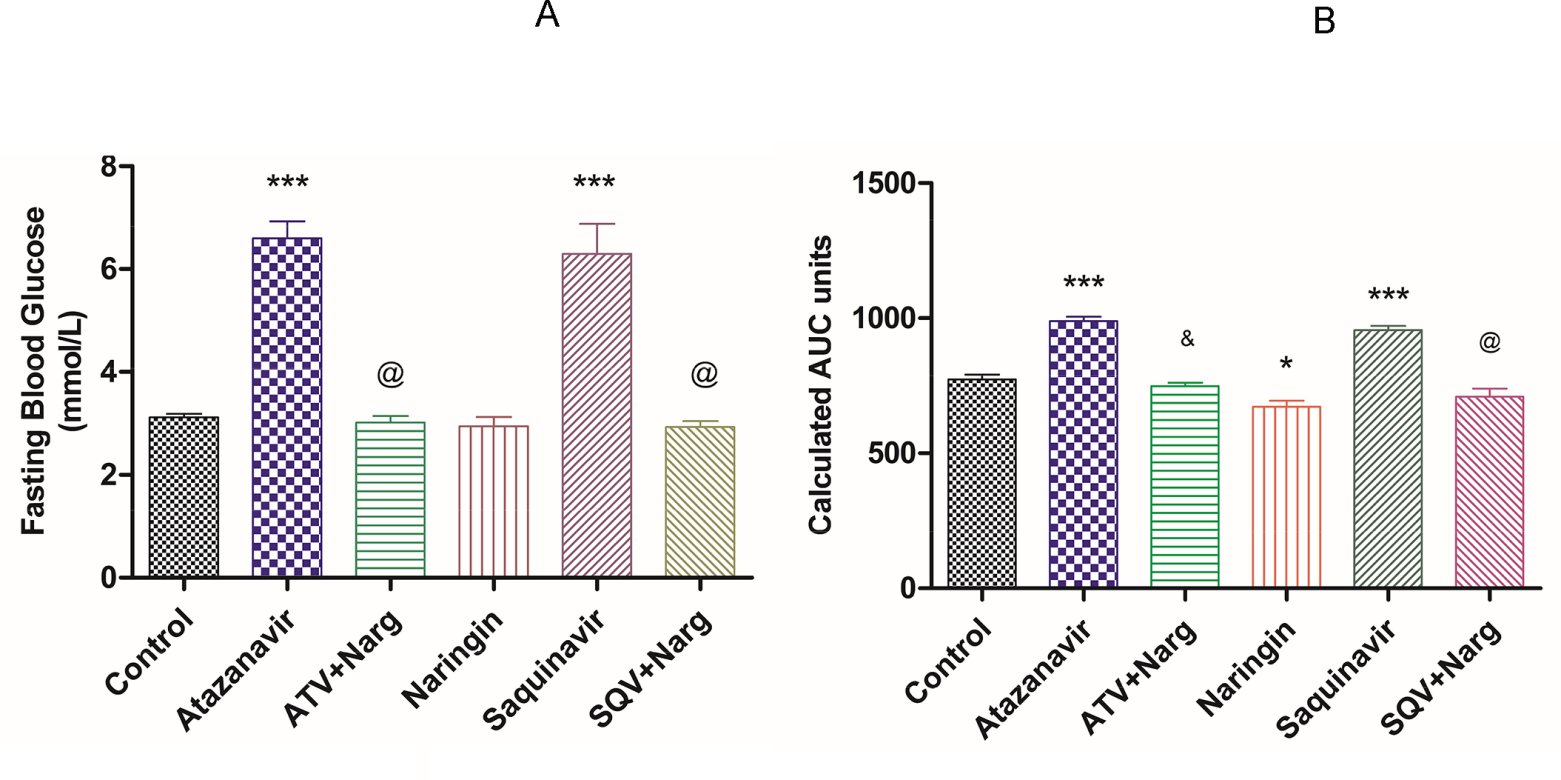
**A**) Fasting Blood Glucose concentrations. ***p < 0.0001 compared to control and ^@^p < 0.0001 compared to ATV or SQV, respectively. **B**) Calculated AUC from Glucose Tolerance Tests- time curves. (*, ***p < 0.05 compared to control, and ^&^, ^@^p < 0.0001 compared to ATV or SQV, respectively).

FBG concentrations and calculated AUC units from GTT were significantly (p < 0.0001) increased in ATV- or SQV-treated rats compared to the control, respectively (Fig 3A and 3B). However, treatment with naringin significantly (p < 0.0001) reduced both FBG concentrations and AUC units in ATV- or SQV-treated rats compared to ATV and SQV-only treated rats, respectively. Naringin treatment had significantly (p < 0.0001) reduced AUC units in GTT compared to the control (Fig 3B).

**Fig 4:**
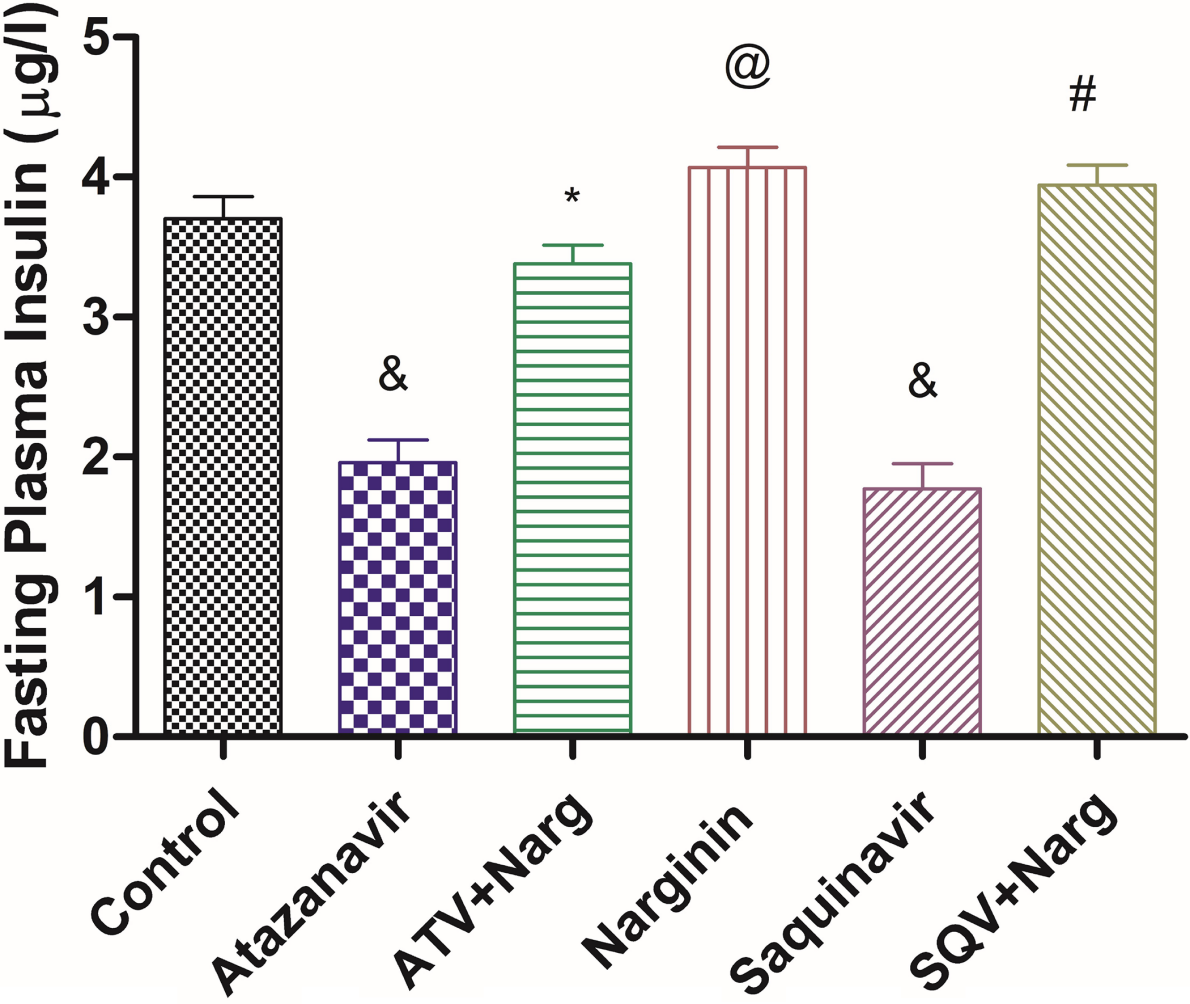
Fasting plasma insulin concentrations. ^&^, ^@^p < 0.0001 compared to control and *, ^#^p < 0.0001 compared to ATV and SQV, respectively.

FPI concentrations were significantly (p < 0.0001) reduced in ATV- or SQV-treated rats compared to control, while concomitant administration of naringin with ATV or SQV significantly (p < 0.0001) increased FPI concentrations compared to ATV- and SQV-only treated animals, respectively (Fig 4). However, naringin-only-treated rats exhibited significantly (p < 0.0001) higher FPI levels compared to the control (Fig 4).

**Fig 5:**
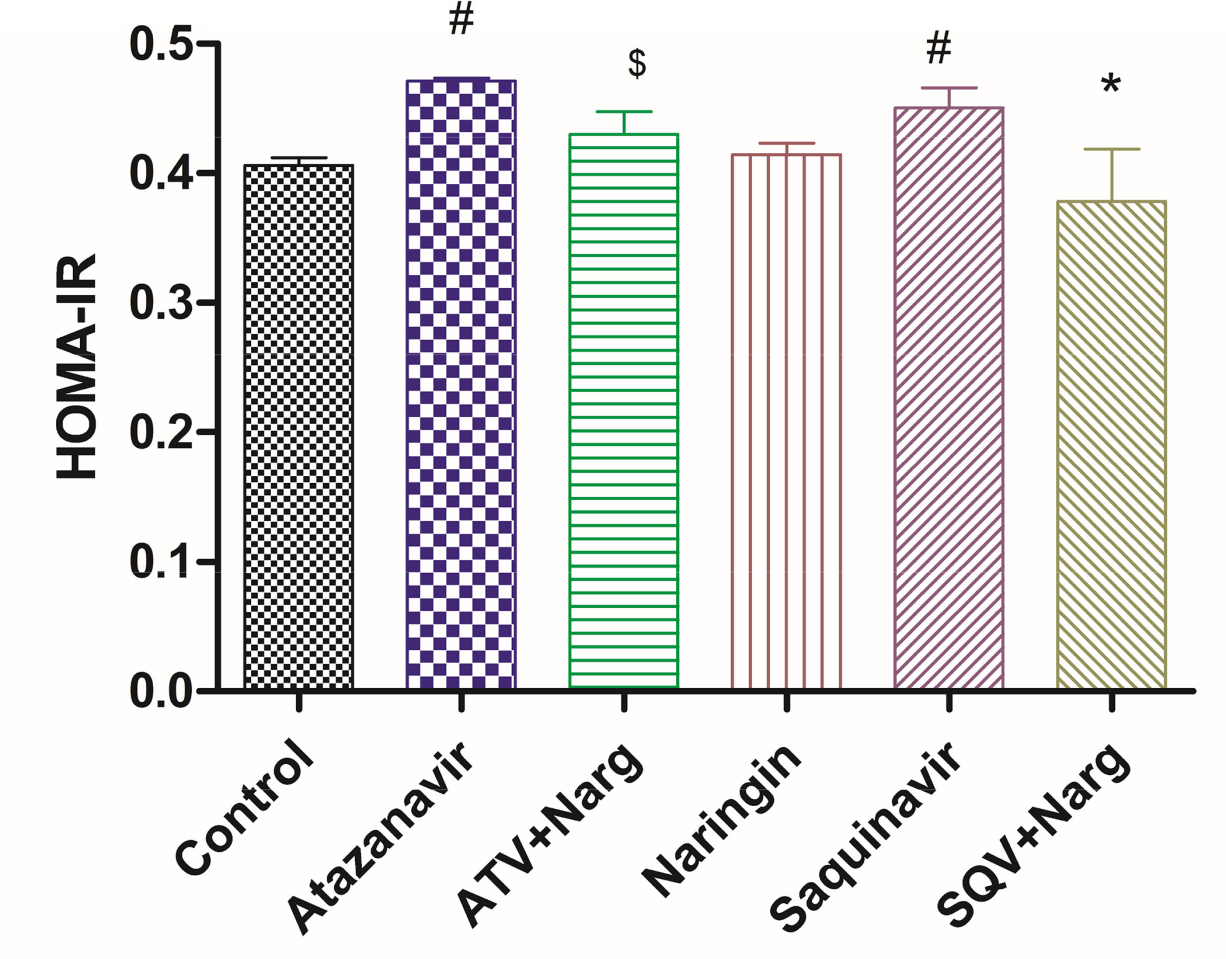
Homeostasis Model Assessment (HOMA) of insulin resistance in PIs and/or naringin treated rats. ^#^p < 0.05 compared to control. ^$,^ *p < 0.05 compared to ATV or SQV, respectively.

Calculated HOMA-IR were significantly (p < 0.05) elevated in ATV- or SQV-treated rats compared to the control (Fig 5). However, naringin significantly (p < 0.05) decreased HOMA-IR values compared to ATV- and SQV-only treated rats, respectively (Fig 5).

### Insulin signaling

**Fig 6:**
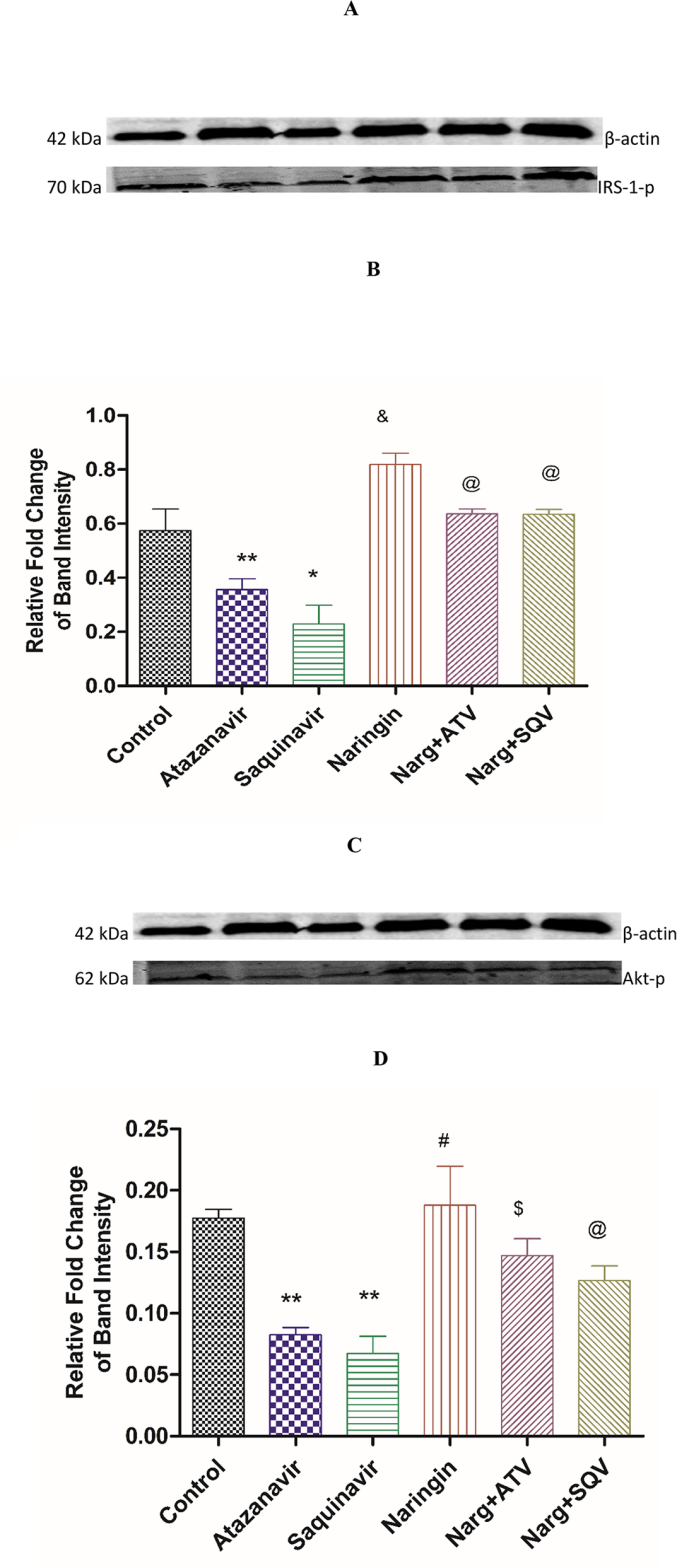
Immunoblots of **A**) Phosphorylated IRS-1 protein expression analyzed by Western blotting against β-actin bands for equal loading and **B**) corresponding densitometry scans. *^,^**p < 0.05 compared to control, ^$^ p < 0.05 compared to ATV and SQV, respectively. **C)** Phosphorylated Akt protein expression analyzed by Western blotting against β-actin bands for equal loading and **D)** corresponding densitometry scans. *^,^**p = 0.05 compared to control, ^$^ p = 0.05 compared to ATV and SQV, respectively.

ATV- or SQV-only-treated rats exhibited significantly (p = 0.0014) decreased expression of phosphorylated IRS-1 proteins compared to controls, respectively, (Fig 6A and 6B). Administration of naringin with either ATV or SQV significantly (p = 0.0014) elevated the levels of phosphorylated IRS-1 compared to ATV- or SQV-only-treated rats, respectively.

Similarly, protein levels of phosphorylated Akt in ATV or SQV-treated rats were significantly (p < 0.05) decreased compared to the control, respectively (Fig 6C and 6D). Naringin treatment significantly increased the levels of phosphorylated Akt compared to ATV or SQV-only treated rats, respectively. Naringin treatment significantly (p < 0.05) increased the expression of phosphorylated IRIS-1 and Akt proteins in non-PI-treated rats compared to controls, respectively.

### Hepatic and pancreatic glucokinase levels

**Fig 7:**
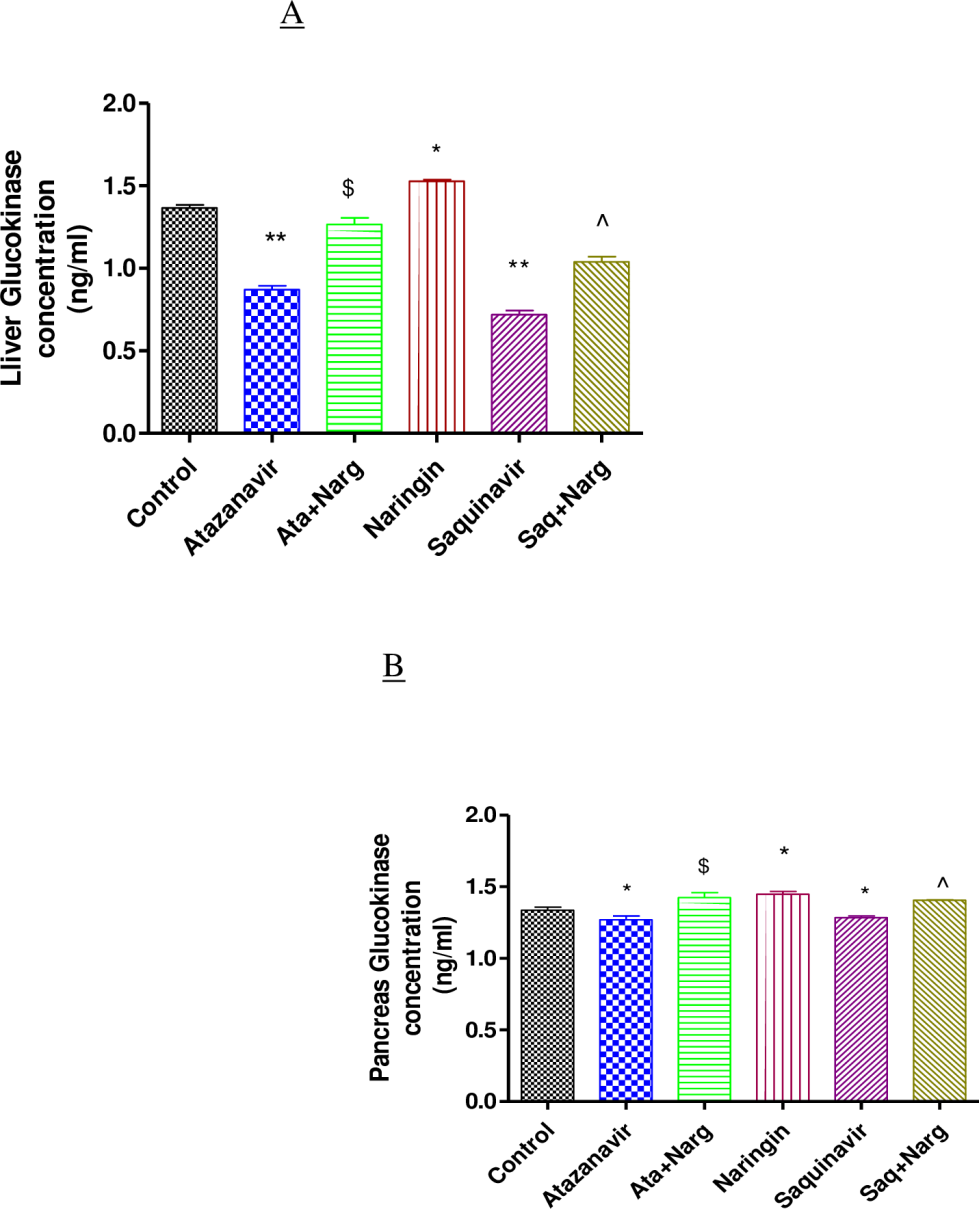
Glucokinase levels in homogenized. **A)** Liver and **B)** Pancreatic tissues. *, ** p < 0.05 compared to control and ^$^, ^ p < 0.05 compared to ATV and SQV, respectively.

ATV or SQV treatment significantly (p < 0.05) reduced glucokinase concentrations in both hepatic and pancreatic tissues compared to controls, respectively (Fig 7). However, naringin treatment significantly (p < 0.05) increased glucokinase concentrations in ATV- or SQV-treated compared to non-treated rats in both hepatic and pancreatic tissues, respectively. Naringin also significantly (p < 0.05) increased hepatic and pancreatic glucokinase content in non-PI-treated animals compared to controls, respectively (Fig 7).

### Pancreatic ATP levels

**Fig 8:**
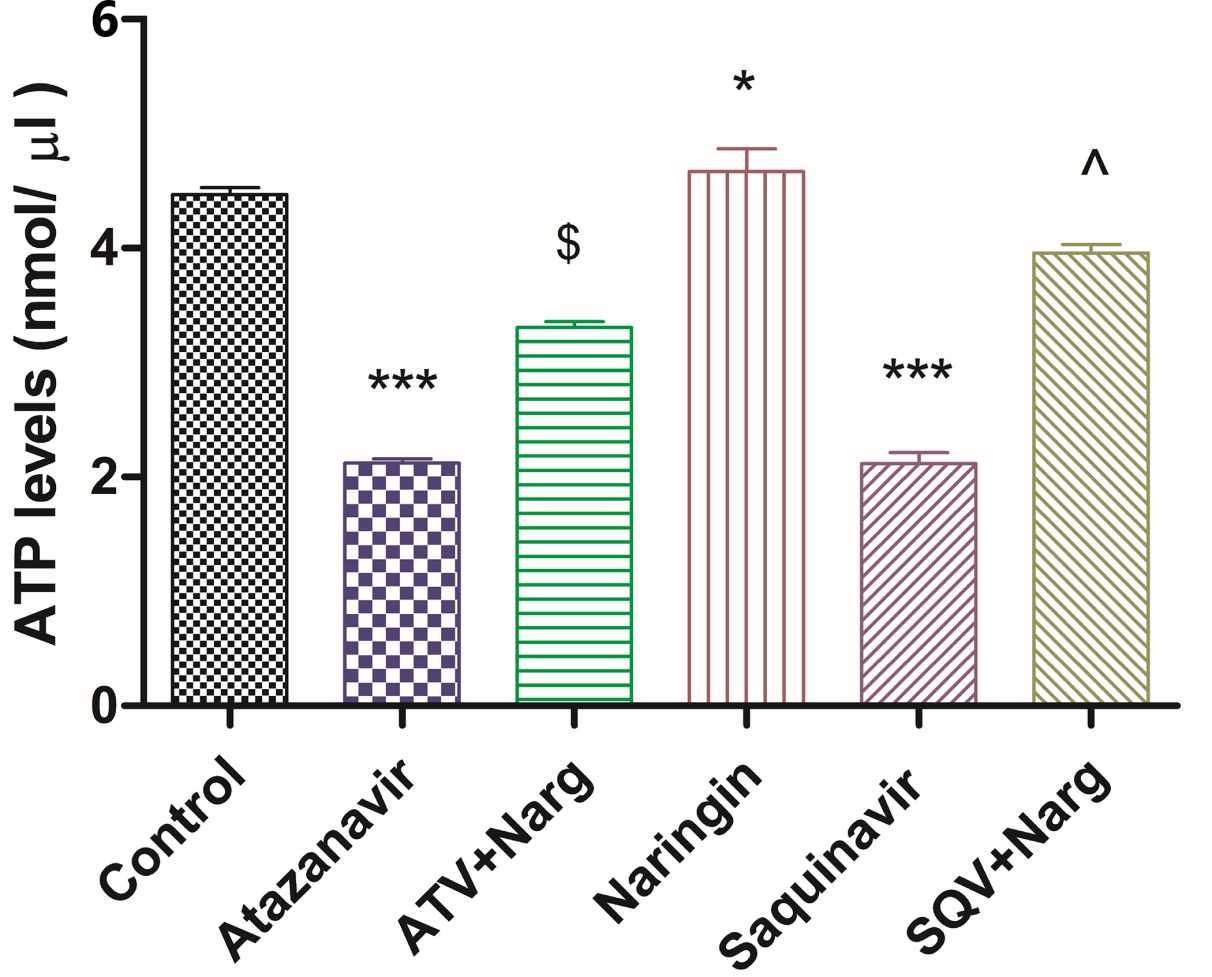
Total ATP concentrations in homogenized pancreatic tissues. *, *** p < 0.05 compared to controls and ^$^, ^ p < 0.05 compared to ATV and SQV, respectively.

Pancreatic ATP levels were significantly (p < 0.0001) reduced in ATV or SQV-only treated rats compared to the control (Fig 8). Nevertheless, treatment with naringin significantly (p < 0.05) increased ATP levels in ATV- or SQV-treated rats compared to ATV- and SQV-treated rats, respectively (Fig 8). However, naringin-treated rats demonstrated significantly (p < 0.05) higher ATP levels compared to the control (Fig 8).

### Pancreatic UCP2 protein expression

**Fig 9:**
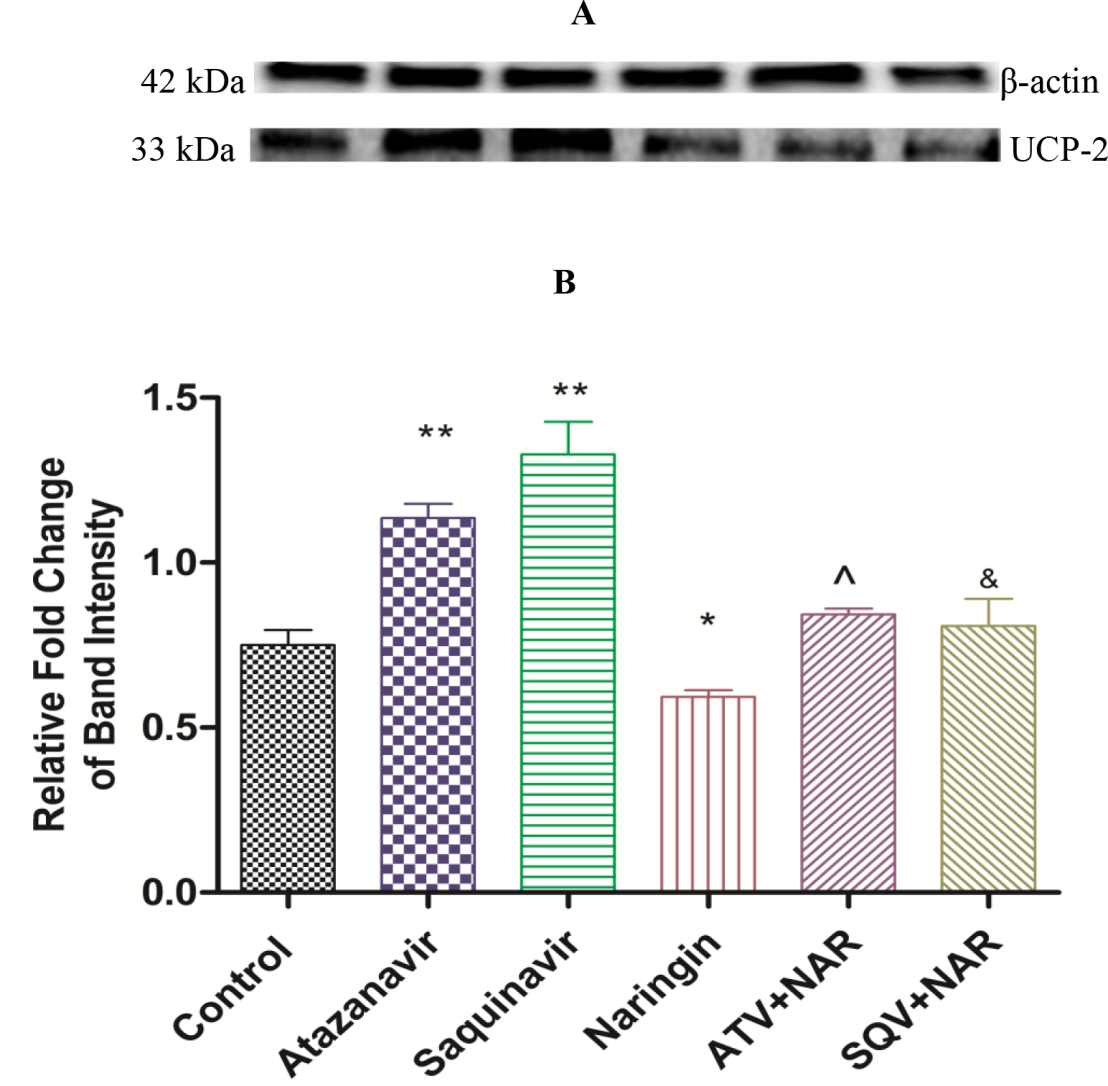
**A**) Western blot analysis of UCP-2 expression normalized to β-actin in the pancreas of the rats treated with ATV and SQV with or without naringin for 56 days and **B**) corresponding densitometry scans expressed as relative fold change in band intensity to control. *^,^ *** p < 0.05 compared to control; ^, ^&^ p < 0.05 compared to ATV and SQV, respectively.

UCP-2 protein levels were assessed using Western blot technique (Fig 9A). ATV- or SQV-treated rats exhibited significantly (p < 0.0001) increased UCP2 expression compared to the control, while in the presence of naringin in either ATV or SQV significantly (p < 0.0001) decreased expression of UCP2 compared to ATV or SQV-only-treated rats. Naringin, however, appeared to have produced a more significant (p < 0.0001) reduction in the expression of UCP2 protein compared to control (Fig 9A and 9B).

### Assessment of apoptosis

**Fig 10:**
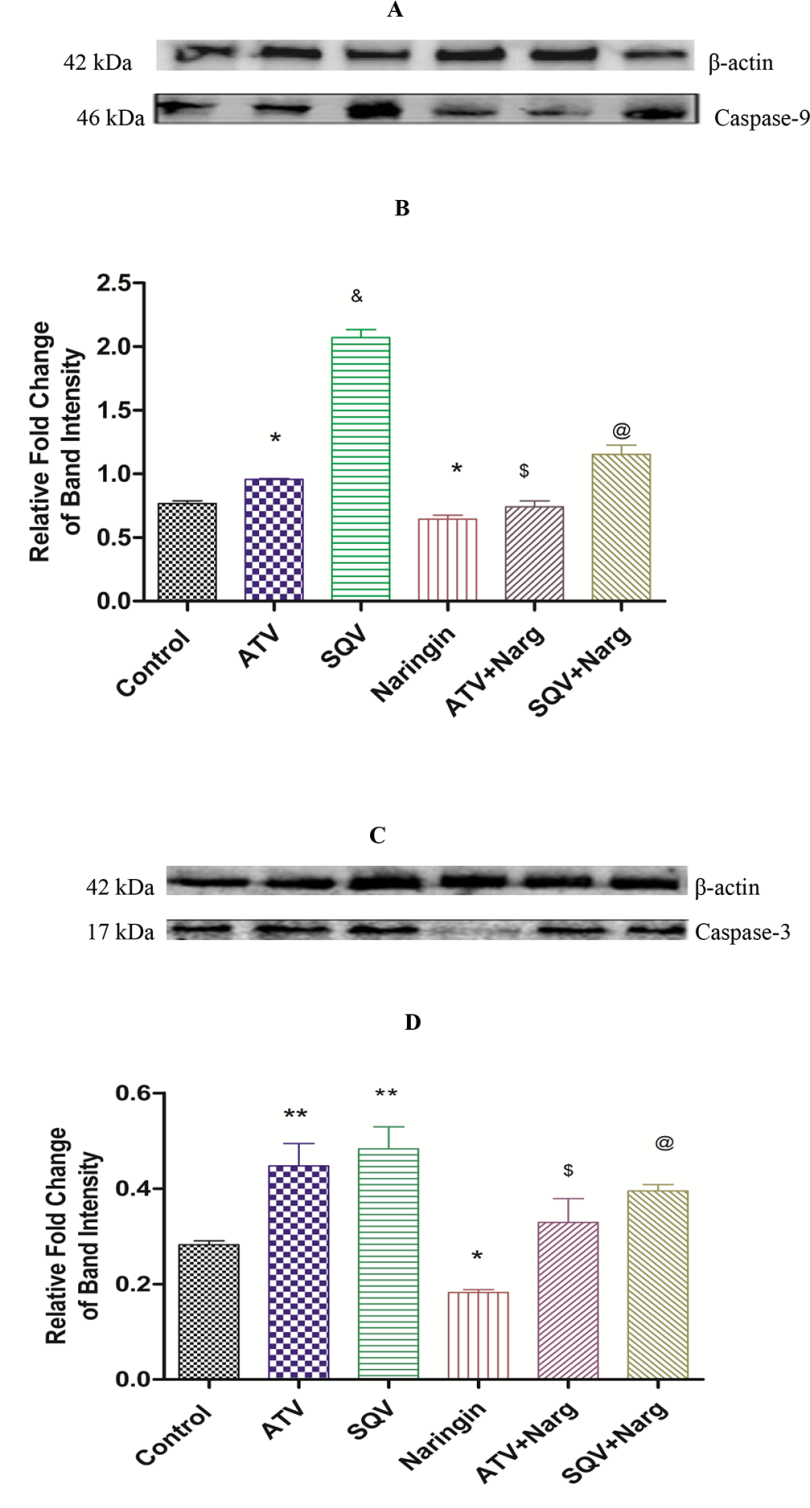
Pancreatic tissue expression of proapoptotic proteins caspases-3 and -9). **A)** Immunoblots of caspase-9 and **C)** caspase-3 protein expressions normalized to β-actin and corresponding densitometry scans (**B** and **D**, respectively) expressed as relative fold change in band intensity to control. *, ** p < 0.05 compared to control; ^$^, ^@^ p < 0.05 compared to ATV and SQV, respectively); ^&^ p < 0.05 compared ATV.

ATV or SQV-only treatment caused significantly (p < 0.0001) increased caspase-3 and -9 protein levels compared to the controls, respectively, while SQV-treated rats exhibited significantly (p < 0.0001) higher levels of caspase-9 compared to ATV-treated rats. Naringin on the other hand, significantly (p < 0.05) decreased both caspase-3 and -9 protein expression compared to ATV- or SQV-only treated rats. However, naringin-only treated rats exhibited significantly (p < 0.05) reduced caspase-3 and -9 protein expressions compared to the controls (Fig 10).

## Discussion

Insulin resistance, glucose intolerance or overt diabetes are known metabolic complications associated with chronic administration of PIs [31]. PIs have previously been reported to induce lipolysis and lipid oxidation [32, 33] elevate intramyocellular lipid accumulation impair adipocyte and glucose metabolism [33–35]. In our study, ATV- or SQV-treated rats exhibited significantly decreased body weights compared to control (Fig 1). However, naringin significantly improved body weights in PI-treated rats, suggesting that naringin prevented proteolytic or lipolytic effects of PIs that could have led to reduced body weights. Alam et al [36] reported that naringin upregulates PPAR-γ protein expression through reducing expression of liver X receptor (LXR), SREBP-1c and SREBP-1a in the liver, and hence improved mitochondrial function and lipid metabolism in diabetic male rats suggesting a role of naringin in upregulating adipogenic transcriptional genes thereby enhancing lipogenesis and/or adipogenesis.

ATV-or SQV-treated rats experienced polydipsia compared to controls which was reduced by naringin treatment (Fig 2). Whereas there was no evidence of increased diuresis in PI-treated animals, it is possible to speculate that PIs caused hyperosmolality following hyperglycemia which stimulated hypothalamic osmoreceptor cells to increase water intake and also led to hypersecretion of Antidiuretic hormone (ADH) [37]. A rare inappropriate ADH secretion has been described in a pediatric patient treated with lopinavir [38]. Naringin treatment, therefore appears to have suppressed the activation of thirst in PI-treated animals. It is intriguing that rats treated with naringin only had significantly increased water intake compared to controls (Fig 2). Naringin has previously been reported to increase ethanol metabolism and also increase plasma ADH in experimental animals [39] although antidiuretic effects of naringin have not been experimentally demonstrated. Our study should have interrogated the presence of glycosuria to corroborate these findings.

Furthermore, ATV- or SQV-treated rats developed glucose intolerance as evidenced by the significantly elevated FBG concentrations and impaired GTT, as well as increased HOMA-IR, compared to the control, respectively (Figs 3 and 5). Although HOMA is designed for diabetic models where there is hyperinsulinemia, in our case, FPI concentrations were significantly reduced in ATV- or SQV-only-treated rats compared to the control (Fig 4) yet there was evidence of increased insulin resistance in rats that were exposed to PIs. This suggests that PIs also cause insulin resistance by extrapancreatic effects. HIV PIs have been reported to impair insulin secretion in the pancreatic β-cells by increasing cellular oxidative stress leading to reduced ATP production [18]. Upregulation of mitochondrial UPC2 has previously been reported to uncouple oxidative phosphorylation and impair the activity ATP synthase leading to reduced ATP production and impaired insulin secretion [40]. In our study, pancreatic expression of UCP2 was significantly increased in either ATV- or SQV-treated animals while naringin treatment significantly reduced the expression of UCP2 in ATV- or SQV-treated rats compared to non ATV or SQV only-treated animals (Fig 9). Naringin treatment further significantly reduced UCP2 expression in non-PI exposed rats compared to control. Furthermore, ATP production was significantly reduced in ATV- or SQV-treated animals compared to controls but this was significantly reversed by treatment with naringin (Fig 8). These results taken in the context of our previous investigations [16] therefore suggest that ATV or SQV increased oxidative stress which upregulated the expression of pancreatic UCP2 leading to uncoupling of oxidative phosphorylation, reduced ATP synthesis and impaired insulin secretion. We have recently described impairment of insulin secretion through increased cellular oxidative stress and ATP depletion by PIs, and further shown that naringin through its antioxidant effects reduced oxidative stress and increased insulin secretion in β-cells exposed to PIs [16]. In this study, naringin treatment significantly increased FPI concentrations in rats that were not exposed to PIs (Fig 4) similarly to our previous findings in vitro [16] suggesting that naringin reduced oxidative stress even in normal rats leading to increased ATP production and increased insulin synthesis in the pancreatic β-cells in normal rats compared to controls.

Furthermore, naringin treatment significantly improved glucose tolerance not only in normal but also in PI-exposed rats compared to the control and PI-treated rats, respectively (Fig 4B). Antihyperglycemic effects of naringin have previously been described [25, 26]. In our study, naringin treatment significantly increased glucokinase protein content in both hepatic and pancreatic tissue compared to either controls or ATV- or SQV-treated animals, respectively (Fig 7). These results therefore corroborate the previously reported antihyperglycemic effects of naringin [41]. Glucokinase catalyses the rate-limiting step in glycolysis which in the liver signals the irreversible trapping of glucose in the storage form as glycogen and in the pancreas marks the initiation of ATP generation and insulin secretion.

Insulin receptor-mediated signal transduction cascade is crucial in the regulation of blood glucose concentrations [42, 43]. Skeletal muscle is accountable for approximately 90% of insulin-induced glucose disposal [6]. Insulin binds and causes activation of tyrosine kinase on its receptor of the β-subunit leading to autophosphorylation of IRS-1 and 1RS-2 [20, 43]. Phosphatidylinositol (4,5)-bisphosphate (PIP_2_) and Phosphatidylinositol (3,4,5)-trisphosphate (PIP_3_) are generated by action of PI- 3-kinase causing phosphorylation of Thr^308^/Ser^437^ by PIP3-dependent protein kinase (PDK) and activation of Akt [20, 44]. The phosphorylated Akt eventually leads to translocation of GLUT 4 to cell membrane to initiate glucose transport [20, 44].

Our finding suggests that ATV or SQV impaired insulin signaling by significantly reducing the expression of both phosphorylated IRS-1 and Akt proteins and that naringin treatment significantly increased the expression of these proteins in PI-treated animals (Fig 6A and 6B). This suggests that ATV or SQV caused insulin resistance and reduced glucose tolerance by impairing insulin signaling and that naringin improved insulin sensitivity in PI-treaded animals leading to improved glucose tolerance. Down-regulation of phosphorylated IRS-1 or Akt proteins, has been shown to inactivate downstream signaling events and subsequently reduce glucose uptake and cause hyperglycemia and peripheral insulin resistance [11, 45].

We have previously shown that PIs are associated with increased activities of caspase -3 and -9 in vitro which was abrogated by naringin [16] and here our data show that ATV or SQV significantly increased pancreatic caspase-3 and -9 protein expressions in vivo (Fig 10). Our results further show that naringin significantly reduced protein expression of these caspases compared to PI-treated or control animals, respectively (Fig 10). Caspases are a family of serine proteases that are critical in apoptosis and caspase-3 and -9 play key regulatory roles in this process [46]. Activation of caspase-9 by SQV was significantly increased compared to ATV (Fig 10B) supporting the well known permissive effects of ATV on metabolic complications generally associated with PI [7]. Our results therefore suggest that ATV and SQV impair pancreatic function by amongst others inducing apoptosis and that naringin perhaps through its antioxidant effects prevents PI-induced apoptosis of the pancreatic cells.

## Conclusion

ATV- and SQV-induce glucose intolerance, reduce insulin secretion and impair insulin signaling in rat skeletal muscles. Naringin treatment ameliorates these metabolic complications by reducing glucose intolerance, insulin resistance and increased insulin sensitivity hence could possess metformin-like effects. Dietary supplementation with naringin could mitigate metabolic complications associated with PI-based antiretroviral therapy.

## Supporting information

S1 File Quantitative values of the result of immunoblot analysis. (TIF)

S2 File Raw data of glucokinase levels in the liver. (TIF)

S3 File Calculated AUC values. (TIF)

S4 File Calculated AUC values. (TIF)

S5 File Fasting blood glucose values. (TIF)

S6 File Average data of water intake. (TIF)

S7 File Data on body weighs. (TIF)

S8 File Raw data of glucokinase levels in the pancreas. (TIF)

S9 File Calculated HOMA values of insulin resistance Fig 5. (TIF)
