# Supplementary figures and images for: Naringin prevents HIV-1 protease inhibitors-induced metabolic complications in vivo
Source: PLoS One. 2017 Nov 9;12(11):e0183355. doi: 10.1371/journal.pone.0183355 (PMC5679664; doi:10.1371/journal.pone.0183355)

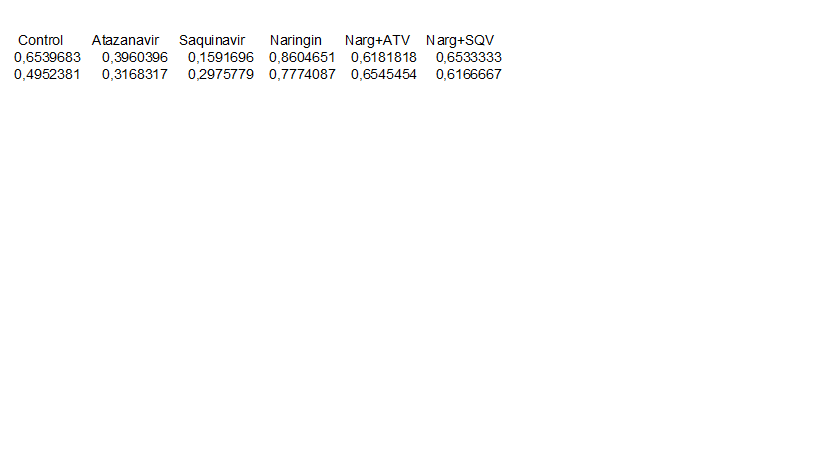

Supplement: S1 File — (TIF) [file pone.0183355.s001.tif]

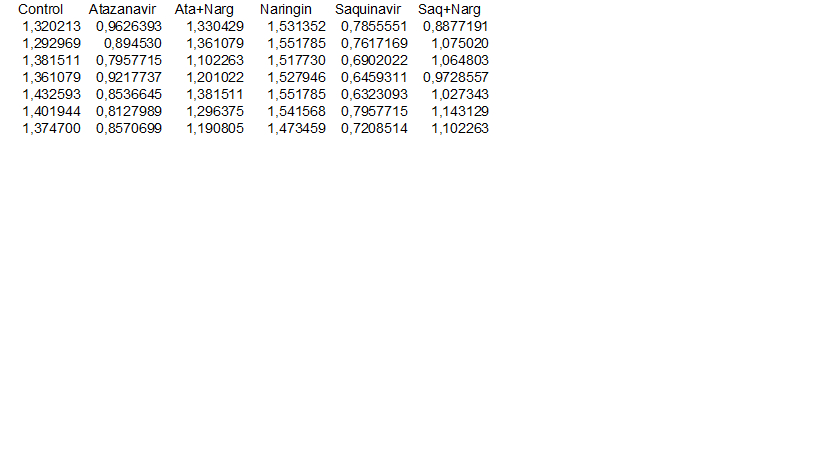

Supplement: S2 File — (TIF) [file pone.0183355.s002.tif]

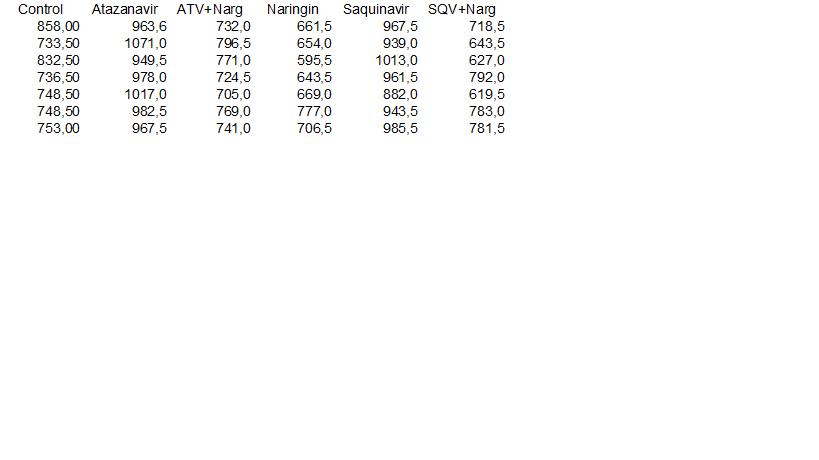

Supplement: S3 File — (TIF) [file pone.0183355.s003.tif]

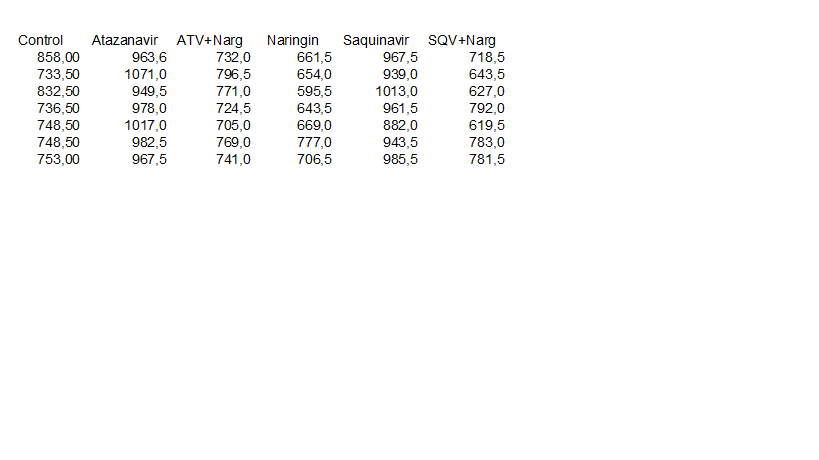

Supplement: S4 File — (TIF) [file pone.0183355.s004.tif]

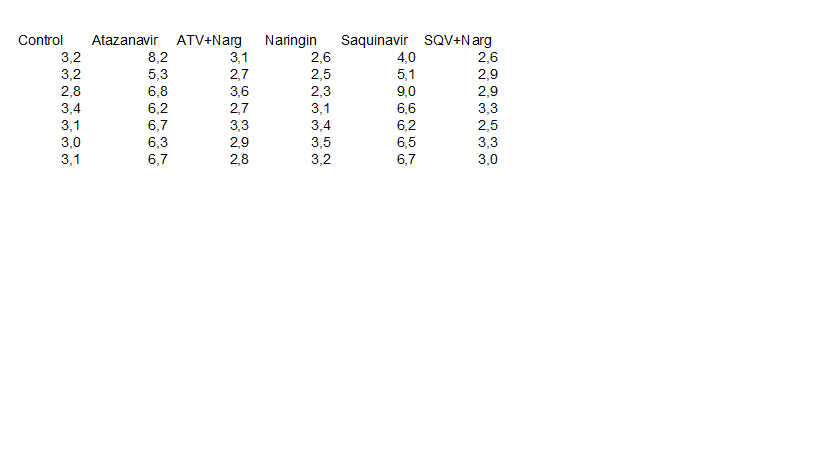

Supplement: S5 File — (TIF) [file pone.0183355.s005.tif]

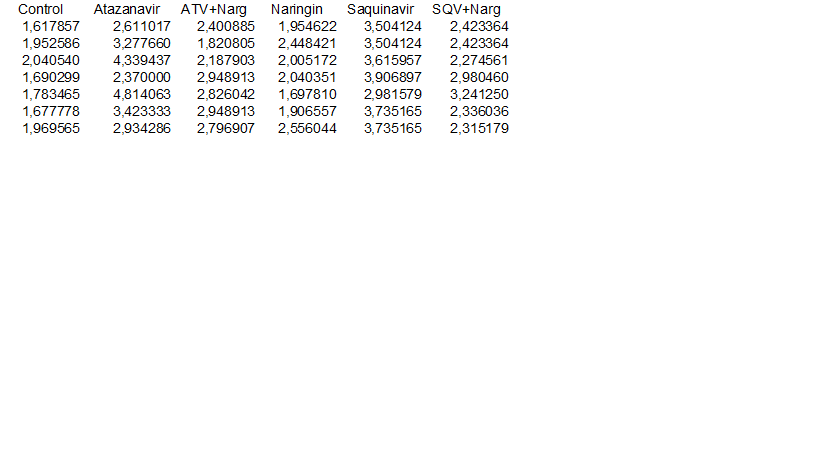

Supplement: S6 File — (TIF) [file pone.0183355.s006.tif]

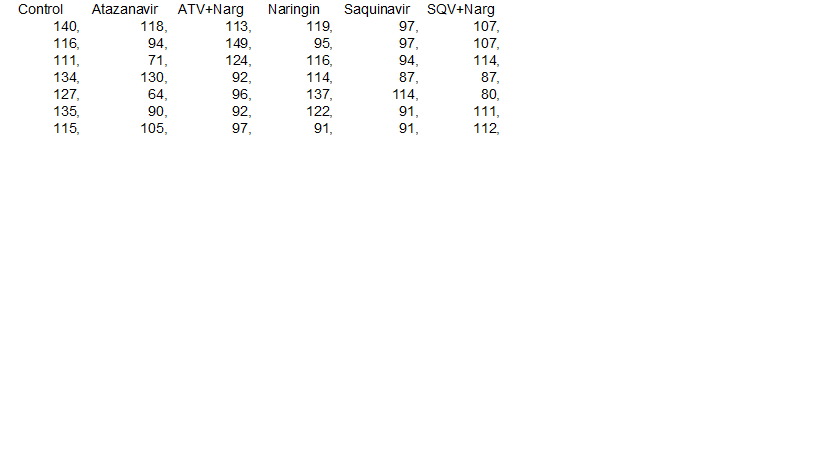

Supplement: S7 File — (TIF) [file pone.0183355.s007.tif]

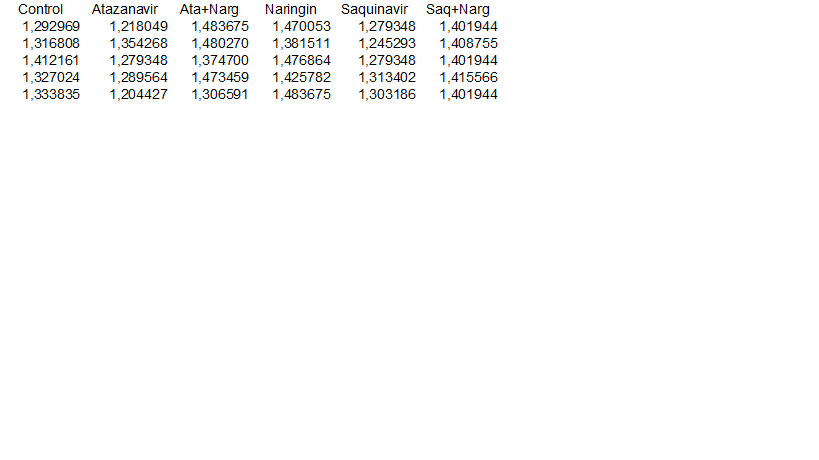

Supplement: S8 File — (TIF) [file pone.0183355.s008.tif]

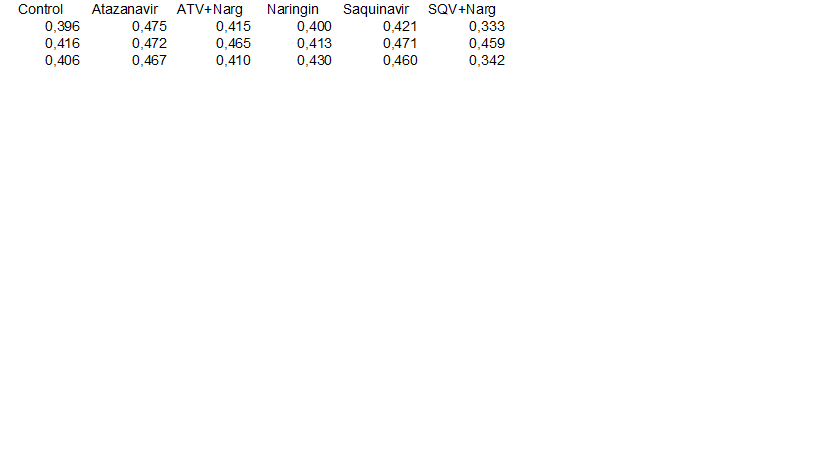

Supplement: S9 File — (TIF) [file pone.0183355.s009.tif]
